# Therapeutic targeting of Aurora Kinase A in advanced prostate cancer

**DOI:** 10.17161/sjm.v2i2.23274

**Published:** 2025-02-07

**Authors:** Maroun Bou Zerdan, Mehmet Asim Bilen, Jindan Yu

**Affiliations:** Department of Hematology and Medical Oncology, Emory University School of Medicine, Atlanta, GA 30322

**Keywords:** prostate cancer, Aurora Kinase, targeted therapy

## Commentary

Prostate cancer (PCa) typically progresses from an androgen-dependent state to a more aggressive form known as castration-resistant prostate cancer (CRPC) following androgen deprivation therapy (ADT). ADT, which includes surgical or medical castration, initially reduces tumor burden by inhibiting androgen receptor (AR) signaling, a critical driver of prostate cancer growth [1, 2]. However, CRPC eventually develops due to several mechanisms that restore or bypass AR signaling. These mechanisms include AR gene amplification, AR mutations, expression of constitutively active AR splice variants, and intratumoral androgen synthesis [1–3]. The molecular mechanisms underlying this transition involve genetic, epigenetic, and hormonal changes that promote cellular plasticity. Key genetic alterations include the loss of tumor suppressor genes such as RB1, TP53, and PTEN, and changes in epigenetic regulators like EZH2, which facilitate the lineage switch from prostate adenocarcinoma to NEPC [4, 5]. Additionally, transcription factors such as ASCL1 and SOX2 play crucial roles in driving neuroendocrine differentiation and maintaining the NE phenotype [6, 7]. The development of NEPC often follows extensive androgen receptor pathway inhibitors, antiandrogen resistance, and a loss of androgen receptor expression. Despite castrate levels of circulating androgens, these adaptations allow continued AR activity, driving tumor progression [1, 2]. A subset of CRPC tumors, however, can become independent of AR signaling and adopt neuroendocrine features, leading to neuroendocrine prostate cancer (NEPC). The emergence of NEPC is associated with resistance to conventional therapies and poor prognosis. NEPC is characterized by the loss of AR expression and the gain of neuroendocrine markers, such as chromogranin A and synaptophysin [4]. This transition is often driven by genetic and epigenetic changes, including the loss of tumor suppressors like TP53 and RB1, the activation of lineage plasticity pathways, with concomitant upregulation of cell cycle drivers including MYCN, PLK1, Cyclin D1, and Aurora kinase A (AURKA) [8–10].

AURKA is a serine/threonine kinase that controls the timing of mitotic entry and spindle formation and promotes cell division. AURKA expression is increased in PCa, and its level is associated with tumor aggressiveness [11–14]. Early genetic studies of Aurora A mutants consistently revealed defects in the formation and regulation of the bipolar spindle during mitosis. A more in-depth examination of Aurora A’s expression, activation, and phosphorylation targets has further clarified its role in a series of earlier cell cycle events that set the stage for proper mitotic progression [15]. Aurora A governs several key processes, including centrosome maturation and separation, bipolar spindle assembly, mitotic entry initiation, chromosome alignment in metaphase, and cytokinesis/abscission. Additionally, the proteolytic degradation of Aurora-A is essential for cells to transition into G1 [15]. AURKA is frequently overexpressed in NEPC and CRPC, contributing to the aggressive nature of these cancers. In patients who developed treatment-induced neuroendocrine PCa, the AURKA gene is amplified in 65% of primary tumors and 86% of metastatic tumors [16, 17]. Increased AURKA not only promotes mitotic spindle formation and cell cycle progression but also interacts with other oncogenic pathways, including those involving MYCN and AR variants [16, 18]. The reciprocal regulation between AURKA and tumor suppressors like NKX3.1 further underscores its role in NEPC pathogenesis [18].

AURKA phosphorylates p53 at specific sites, leading to its destabilization and degradation. For instance, AURKA phosphorylates p53 at Ser315, which facilitates its ubiquitination by Mdm2 and subsequent proteolysis. This degradation of [19] reduces its tumor suppressor functions, including cell cycle arrest and apoptosis, thereby allowing cancer cells to proliferate despite the presence of functional p53.

Additionally, AURKA can phosphorylate p53 at Ser215, which abrogates p53’s DNA binding and transactivation activity, further impairing its ability to induce the expression of downstream target genes such as p21 and PTEN [20]. This inhibition of p53’s transcriptional activity contributes to the survival and proliferation of cancer cells under therapeutic pressure.

Moreover, AURKA disrupts the interaction between p53 and its coactivators, such as hnRNPK, by phosphorylating hnRNPK, which further diminishes p53’s transcriptional activity [21]. This multifaceted inhibition of p53 by AURKA allows cancer cells to bypass p53-mediated growth suppression and apoptosis, contributing to drug resistance.

In the context of NEPC, AURKA overexpression is often observed and is associated with the aggressive behavior of these tumors. The ability of AURKA to inactivate p53 through multiple mechanisms underscores its role in overcoming drug resistance and promoting NEPC progression [22, 23].

**Figure 1.**
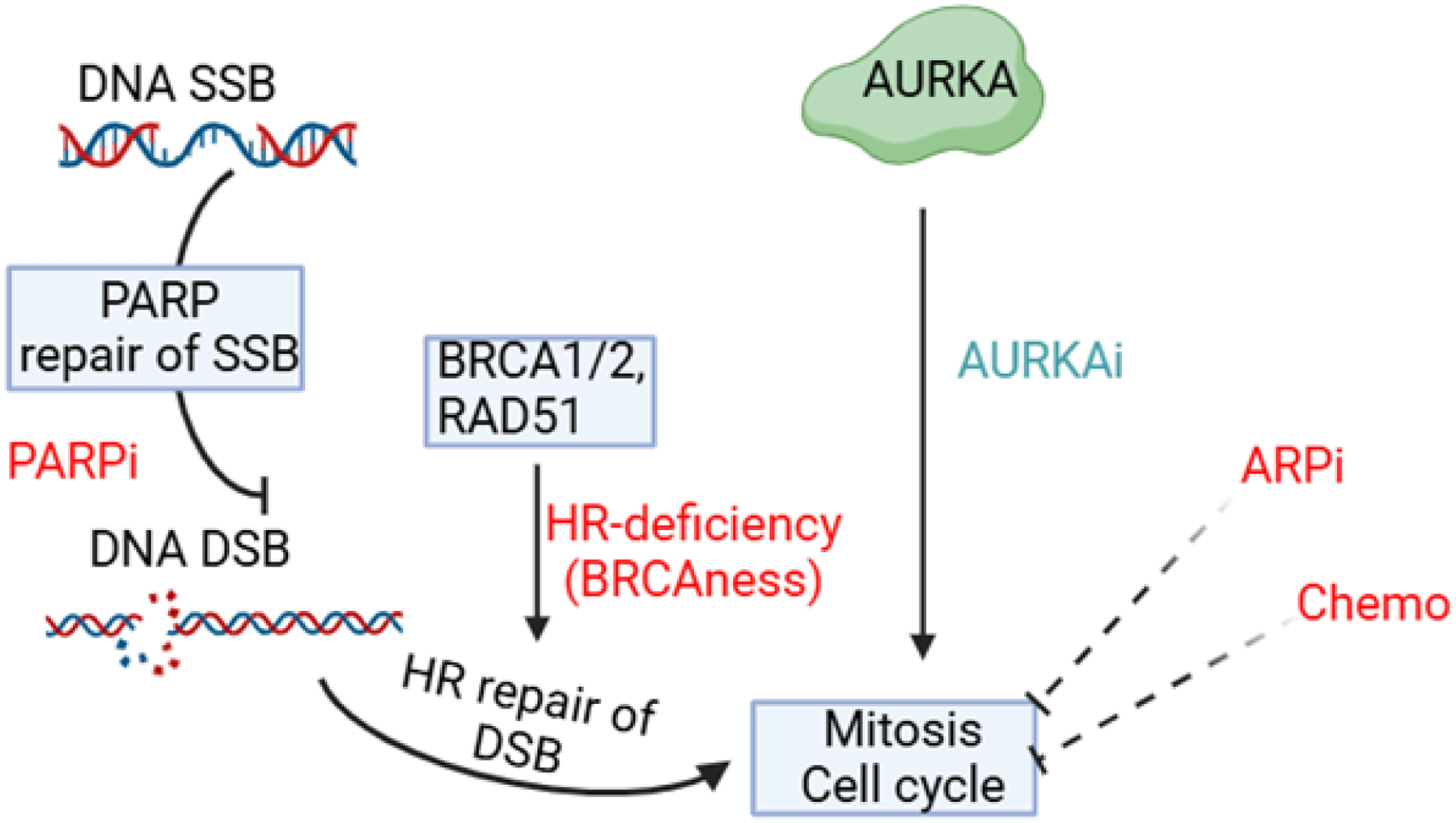
AURKA activity critically promotes mitosis and the cell cycle. AURKA inhibition (AURKAi) might be synthetically lethal with HR deficiency and work synergistically with other DNA-damaging therapeutics such as PARPi, ARPi, and chemotherapy (Chemo) to inhibit PCa growth. *SSB: single strand break. DSB: double-strand break*.

In a recent study, Gritsina et al. reported that C-X-C chemokine receptor type 7 (CXCR7) is upregulated in NEPC [24], following up their earlier study demonstrating a causative role of CXCR7 in driving enzalutamide-resistant PCa and thus, CRPC progression [25]. They showed that CXCR7 promotes tumor growth and proliferation by activating downstream signaling pathways, such as AURKA [24]. Specifically, CXCR7 recruits β-arrestin (ARRB2) and forms a complex that activates AURKA, a key regulator of mitosis. They confirmed in PCa patient datasets that AURKA signal transduction is positively correlated with CXCR7 expression. Further, the study tested the AURKA inhibitor alisertib and revealed that alisertib abolishes CXCR7-driven PCa growth in cell lines as well as animal models [24]. The authors thus proposed AURKA as a critical therapeutic target for CXCR7-high CRPC and NEPC patients. However, a Phase II clinical trial of alisertib in CRPC/NEPC patients failed to meet its primary endpoint due to drug toxicity and patient variability despite significant clinical benefits in a small subset of four patients with high AURKA expression [26]. Further evaluation of the drug might benefit from molecular biomarkers to preselect patients with AURKA activation and/or drug combinations to lower the dose and reduce toxicity. Alternative AURKA inhibitors are currently being investigated. Some of these include the evaluation of AL8326 in ≥2nd line small cell lung cancer, a study of Tinengotinib (TT-00420) in combination with standard treatments in people with prostate cancer, and JAB-2485 activity in adult patients with advanced solid tumors [27–29]. Notably, inhibiting AURKA is synthetically lethal with the loss of RB1 or p53 tumor suppressor genes due to AURKA’s vital role in driving the cell cycle in these tumors [30, 31]. Likewise, cells harboring a high mutational burden, as often seen in tumors with defective homologous recombination (HR), might become increasingly reliant on AURKA activity (Figure 1). In these cells, AURKA might help bypass cell cycle checkpoints, thereby enabling continued replication and tumor growth despite DNA damage [32]. As such, AURKA activity has been closely linked to tumorigenesis and the expression of genes associated with metastasis [33]. Inhibition of AURKA activity has been shown to cause delayed mitotic progression, mitotic failure, and ultimately, cell death [33]. These findings suggest that AURKA could serve as a promising therapeutic target, particularly in tumors with AURKA amplification or up-regulation, such as CRPC and NEPC. Furthermore, AURKA overexpression may contribute to resistance to therapies that target DNA repair pathways, such as PARP inhibitors like Olaparib, as well as resistance to androgen receptor pathway inhibitors (ARPi) and chemotherapy [34]. AURKA may be essential for driving cell cycle progression in PCa cells exposed to DNA-damaging therapeutics, including PARPi, ARPi, and chemotherapeutic agents [34]. To this end, it will be essential to evaluate the frequency of genomic alterations, including AURKA amplification or overexpression, RB1 loss and HR gene mutations in CRPC and NEPC tumors, and their association with therapeutic (PARPi, ARPi, and Chemotherapy) responses. If positive correlations were discovered, it would support targeting AURKA to overcome therapeutic resistance and improve treatment outcomes for these aggressive and treatment-refractory cancers.

In summary, prostate cancer progresses to CRPC through mechanisms that sustain AR signaling despite androgen deprivation. A subset of CRPC tumors further evolves into NEPC, characterized by AR independence and neuroendocrine differentiation, driven by genetic and epigenetic alterations [1, 4, 35]. In prostate cancer and CRPC with neuroendocrine traits, CXCR7 activates AURKA, promoting NEPC growth. Targeting AURKA, either directly or through its upstream regulators like CXCR7, represents a promising therapeutic strategy for managing NEPC. AURKA is overexpressed in PCa, particularly in CRPC and NEPC, where it promotes cell division, tumor aggressiveness, and metastasis. Targeting AURKA may provide a promising therapeutic strategy, especially in combination with PARPi like Olaparib, to improve outcomes in HR-deficient PCa, including cases with mutations beyond BRCA1/2. Similarly, AURKA inhibition in patients with wild-type HR genes could be utilized, as it has been shown to regulate the stability of Myc proteins, which in turn influence the expression of HR-related genes. This strategy could enhance the effectiveness of PARPi in HR-proficient PCa, particularly in tumors with high Myc expression, by inducing vulnerabilities similar to those seen in BRCA-mutant cancers. Last, AURKA inhibitors might improve outcomes of standard-of-care of metastatic PCa that uses AR pathway inhibitors, such as Enzalutamide, by intensifying AR inhibition, increasing DNA-damage-related cell death, and preventing the development of escape mechanisms, such as AR variants.

## References

[1] Feng Q, He B: Androgen receptor signaling in the development of castration-resistant prostate cancer. Frontiers in oncology 2019, 9:858. 31552182 10.3389/fonc.2019.00858 PMC6738163

[2] Fontana F, Limonta P: Dissecting the hormonal signaling landscape in castration-resistant prostate cancer. Cells 2021, 10(5):1133. 34067217 10.3390/cells10051133 PMC8151003

[3] Coutinho I, Day TK, Tilley WD, Selth LA: Androgen receptor signaling in castration-resistant prostate cancer: a lesson in persistence. Endocrine-related cancer 2016, 23(12): T179–T197. 27799360 10.1530/ERC-16-0422

[4] Imamura J, Ganguly S, Muskara A, Liao RS, Nguyen JK, Weight C, Wee CE, Gupta S, Mian OY: Lineage plasticity and treatment resistance in prostate cancer: the intersection of genetics, epigenetics, and evolution. Front Endocrinol (Lausanne) 2023, 14:1191311. doi:10.3389/fendo.2023.1191311. 37455903 PMC10349394

[5] Maylin ZR, Smith C, Classen A, Asim M, Pandha H, Wang Y: Therapeutic Exploitation of Neuroendocrine Transdifferentiation Drivers in Prostate Cancer. Cells 2024, 13(23):1999. 39682746 10.3390/cells13231999 PMC11639977

[6] Nie J, Zhang P, Liang C, Yu Y, Wang X: ASCL1-mediated ferroptosis resistance enhances the progress of castration-resistant prostate cancer to neurosecretory prostate cancer. Free Radical Biology and Medicine 2023, 205:318–331. doi:10.1016/j.freeradbiomed.2023.06.006:37355053

[7] Rodarte KE, Nir Heyman S, Guo L, Flores L, Savage TK, Villarreal J, Deng S, Xu L, Shah RB, Oliver TG, : Neuroendocrine Differentiation in Prostate Cancer Requires ASCL1. Cancer Res 2024, 84(21):3522–3537. doi:10.1158/0008-5472.Can-24-1388. 39264686 PMC11534540

[8] Tang F, Xu D, Wang S, Wong CK, Martinez-Fundichely A, Lee CJ, Cohen S, Park J, Hill CE, Eng K: Chromatin profiles classify castration-resistant prostate cancers suggesting therapeutic targets. Science 2022, 376(6596):eabe1505. 35617398 10.1126/science.abe1505 PMC9299269

[9] Beltran H, Rickman DS, Park K, Chae SS, Sboner A, MacDonald TY, Wang Y, Sheikh KL, Terry S, Tagawa ST: Molecular characterization of neuroendocrine prostate cancer and identification of new drug targets. Cancer Discovery 2011, 1(6):487–495. 22389870 10.1158/2159-8290.CD-11-0130 PMC3290518

[10] Ku SY, Rosario S, Wang Y, Mu P, Seshadri M, Goodrich ZW, Goodrich MM, Labbé DP, Gomez EC, Wang J: Rb1 and Trp53 cooperate to suppress prostate cancer lineage plasticity, metastasis, and antiandrogen resistance. Science 2017, 355(6320):78–83. 28059767 10.1126/science.aah4199 PMC5367887

[11] Buschhorn HM, Klein RR, Chambers SM, Hardy MC, Green S, Bearss D, Nagle RB: Aurora-A over-expression in high-grade PIN lesions and prostate cancer. Prostate 2005, 64(4):341–346. doi:10.1002/pros.20247:15754349

[12] Kivinummi K, Urbanucci A, Leinonen K, Tammela TLJ, Annala M, Isaacs WB, Bova GS, Nykter M, Visakorpi T: The expression of AURKA is androgen regulated in castration-resistant prostate cancer. Sci Rep 2017, 7(1):17978. doi:10.1038/s41598-017-18210-3. 29269934 PMC5740165

[13] Lee EC, Frolov A, Li R, Ayala G, Greenberg NM: Targeting Aurora kinases for the treatment of prostate cancer. Cancer Res 2006, 66(10):4996–5002. doi:10.1158/0008-5472.CAN-05-2796:16707419

[14] Moore ML, Teitell MA, Kim Y, Watabe T, Reiter RE, Witte ON, Dubey P: Deletion of PSCA increases metastasis of TRAMP-induced prostate tumors without altering primary tumor formation. Prostate 2008, 68(2):139–151. doi:10.1002/pros.20686:18044730

[15] Nikonova AS, Astsaturov I, Serebriiskii IG, Dunbrack RL, Golemis EA: Aurora A kinase (AURKA) in normal and pathological cell division. Cellular and Molecular Life Sciences 2013, 70:661–687. 22864622 10.1007/s00018-012-1073-7 PMC3607959

[16] Beltran H, Rickman DS, Park K, Chae SS, Sboner A, MacDonald TY, Wang Y, Sheikh KL, Terry S, Tagawa ST, : Molecular characterization of neuroendocrine prostate cancer and identification of new drug targets. Cancer Discov 2011, 1(6):487–495. doi:10.1158/2159-8290.CD-11-0130. 22389870 PMC3290518

[17] Mosquera JM, Beltran H, Park K, MacDonald TY, Robinson BD, Tagawa ST, Perner S, Bismar TA, Erbersdobler A, Dhir R : Concurrent AURKA and MYCN gene amplifications are harbingers of lethal treatment-related neuroendocrine prostate cancer. Neoplasia 2013, 15(1):1–10. doi:10.1593/neo.121550. 23358695 PMC3556934

[18] Sooreshjani MA, Kamra M, Zoubeidi A, Shah K: Reciprocal deregulation of NKX3.1 and AURKA axis in castration-resistant prostate cancer and NEPC models. J Biomed Sci 2021, 28(1):68. doi:10.1186/s12929-021-00765-z. 34625072 PMC8499580

[19] Katayama H, Sasai K, Kawai H, Yuan Z-M, Bondaruk J, Suzuki F, Fujii S, Arlinghaus RB, Czerniak BA, Sen S: Phosphorylation by aurora kinase A induces Mdm2-mediated destabilization and inhibition of p53. Nature Genetics 2004, 36(1):55–62. 14702041 10.1038/ng1279

[20] Liu Q, Kaneko S, Yang L, Feldman RI, Nicosia SV, Chen J, Cheng JQ: Aurora-A abrogation of p53 DNA binding and transactivation activity by phosphorylation of serine 215. Journal of Biological Chemistry 2004, 279(50):52175–52182. 15469940 10.1074/jbc.M406802200

[21] Hsueh K-W, Fu S-L, Huang C-YF, Lin C-H: Aurora-A phosphorylates hnRNPK and disrupts its interaction with p53. FEBS letters 2011, 585(17):2671–2675. 21821029 10.1016/j.febslet.2011.07.031

[22] Gritsina G, Fong K-w, Lu X, Lin Z, Xie W, Agarwal S, Lin D, Schiltz GE, Beltran H, Corey E: Chemokine receptor CXCR7 activates Aurora Kinase A and promotes neuroendocrine prostate cancer growth. The Journal of Clinical Investigation 2023, 133(15). 10.1172/JCI166248 PMC10378179 37347559

[23] Sooreshjani MA, Kamra M, Zoubeidi A, Shah K: Reciprocal deregulation of NKX3. 1 and AURKA axis in castration-resistant prostate cancer and NEPC models. Journal of Biomedical Science 2021, 28:1–18. 34625072 10.1186/s12929-021-00765-z PMC8499580

[24] Gritsina G, Fong KW, Lu X, Lin Z, Xie W, Agarwal S, Lin D, Schiltz GE, Beltran H, Corey E : Chemokine receptor CXCR7 activates Aurora Kinase A and promotes neuroendocrine prostate cancer growth. J Clin Invest 2023, 133(15). doi:10.1172/JCI166248. PMC10378179 37347559

[25] Li S, Fong KW, Gritsina G, Zhang A, Zhao JC, Kim J, Sharp A, Yuan W, Aversa C, Yang XJ : Activation of MAPK Signaling by CXCR7 Leads to Enzalutamide Resistance in Prostate Cancer. Cancer Res 2019, 79(10):2580–2592. doi:10.1158/0008-5472.CAN-18-2812. 30952632 PMC6522281

[26] Beltran H, Oromendia C, Danila DC, Montgomery B, Hoimes C, Szmulewitz RZ, Vaishampayan U, Armstrong AJ, Stein M, Pinski J: A phase II trial of the aurora kinase an inhibitor alisertib for patients with castration-resistant and neuroendocrine prostate cancer: efficacy and biomarkers. Clinical Cancer Research 2019, 25(1):43–51. 30232224 10.1158/1078-0432.CCR-18-1912 PMC6320304

[27] Florou V, Orr D, Morton A, Cheng Y, Yuan P, Sun Y, Tang X, Liu S, Gadgeel S: 689TiP A phase I/IIa trial of Aurora-A inhibitor (JAB-2485) in adult patients with advanced solid tumors. Annals of Oncology 2024, 35:S533–S534.

[28] Goldman J, Rosen L, Kung A, Romero A, Qazi I, Dokainish H, Letrent S, Slamon D: 687TiP A phase I, first in human study of TORL-4–500 in patients with advanced cancer. Annals of Oncology 2024, 35:S533.

[29] Hu X, Lu Y, Wang X, Huang Y, Wang F: 688TiP Phase I dose escalation trial to evaluate the safety and preliminary efficacy of ACR246, an innovative 5T4-antibody drug conjugate (ADC), in patients (pts) with advanced solid tumors. Annals of Oncology 2024, 35:S533.

[30] Gong X, Du J, Parsons SH, Merzoug FF, Webster Y, Iversen PW, Chio L-C, Van Horn RD, Lin X, Blosser W: Aurora A kinase inhibition is synthetic lethal with loss of the RB1 tumor suppressor gene. Cancer Discovery 2019, 9(2):248–263. 30373917 10.1158/2159-8290.CD-18-0469

[31] Dar AA, Belkhiri A, Ecsedy J, Zaika A, El-Rifai W: Aurora kinase A inhibition leads to p73-dependent apoptosis in p53-deficient cancer cells. Cancer research 2008, 68(21):8998–9004. 18974145 10.1158/0008-5472.CAN-08-2658 PMC2587495

[32] Milletti G, Colicchia V, Cecconi F: Cyclers’ kinases in cell division: from molecules to cancer therapy. Cell Death & Differentiation 2023, 30(9):2035–2052. 37516809 10.1038/s41418-023-01196-z PMC10482880

[33] Zheng D, Li J, Yan H, Zhang G, Li W, Chu E, Wei N: Emerging roles of Aurora-A kinase in cancer therapy resistance. Acta Pharmaceutica Sinica B 2023, 13(7):2826–2843. 37521867 10.1016/j.apsb.2023.03.013 PMC10372834

[34] Hirst J, Godwin AK: AURKA inhibition mimics BRCAness. Aging (Albany NY) 2017, 9(9):1945. 28898201 10.18632/aging.101291 PMC5636662

[35] Liu S, Alabi BR, Yin Q, Stoyanova T: Molecular mechanisms underlying the development of neuroendocrine prostate cancer. In: Seminars in Cancer Biology: 2022: Elsevier; 2022: 57–68. 10.1016/j.semcancer.2022.05.007 35597438

